# Social support and future self-continuity across clinical transition phases in master of nursing specialist students: a longitudinal cross-lagged panel study based on Schlossberg’s Transition Theory

**DOI:** 10.3389/fpsyg.2026.1822682

**Published:** 2026-05-20

**Authors:** Jialin Lv, Jinxin Cui, Junhui Dong, Xinqi Guo, Lixiu Zhang, Lirong Yuan

**Affiliations:** 1 Department of Nursing, College of Medical Science, Huzhou University, Huzhou, Zhejiang, China; 2 Department of Medical and Nursing, Luzhong Vocational School, Binzhou, Shandong, China; 3 Department of Pulmonary and Critical Care Medicine, First Hospital of Shanxi Medical University, Taiyuan, Shanxi, China; 4 NHC Key Laboratory of Pneumoconiosis, First Hospital of Shanxi Medical University, Taiyuan, Shanxi, China; 5 Shanxi Key Laboratory of Chronic Respiratory Diseases and Pneumoconiosis, First Hospital of Shanxi Medical University, Taiyuan, Shanxi, China

**Keywords:** cross-lagged model, future self-continuity, latent growth model, nursing master, professional degree, social support

## Abstract

**Background:**

Future self-continuity (FSC) is critical to professional identity and career persistence among Master of Nursing Specialist (MNS) students, yet the dynamic interplay between social support and FSC across clinical transition phases remains unclear.

**Objective:**

Guided by Schlossberg’s Transition Theory, this study examined the developmental trajectories of social support and FSC, as well as their phase-dependent, asymmetric temporal relationships, across three transition phases: Moving In (enrollment), Moving Through (early internship), and Moving Out (internship completion).

**Methods:**

A three-wave longitudinal study was conducted with 270 MNS students from multiple institutions in Zhejiang Province, China, who were assessed at enrollment (T1), early internship (T2), and internship completion (T3). Social support and FSC were measured using the SSRS and FSCQ, respectively. Latent growth curve modeling (LGCM) and cross-lagged panel modeling (CLPM) were employed for data analysis.

**Results:**

A total of 263 participants completed all three waves. Social support remained stable (*s* = 0.03, *p* = 0.752), while FSC declined significantly (*s* = −1.576, *p* < 0.001). Baseline FSC showed a positive longitudinal association with changes in social support (*β* = 0.462, *p* < 0.001), but not vice versa (*β* = 0.034, *p* = 0.726). Cross-lagged results showed that FSC demonstrated temporal precedence over subsequent social support across both phases (T1–T2: *β* = 0.252; T2–T3: *β* = 0.109; both *p* < 0.05), whereas social support showed a longitudinal association with FSC only during the pre-clinical phase (T1–T2: *β* = 0.183, *p* < 0.05) and not during the internship phase (T2–T3: *β* = −0.012, *p* > 0.05).

**Conclusion:**

Future self-continuity is more vulnerable to clinical transition challenges than social support and demonstrates a sustained temporal precedence over subsequent social support. The longitudinal effect of social support on FSC is phase-dependent, attenuating as students transition into clinical practice. Interventions should prioritize early FSC cultivation during enrollment and progressively foster internal psychological resources throughout clinical training.

## 1 Introduction

As a core component of the global healthcare system, the quality and effectiveness of care are closely linked to the health and well-being of all people. Nurses are not only providers of daily medical services, but also the backbone of public health emergency response, chronic disease management, and health promotion. High-level nursing talent plays a key role in promoting the industry’s professional and refined development (Van Hecke et al., 2025). However, the structural shortage of global nursing human resources has become a bottleneck restricting the upgrade of the medical system, especially in the field of high-level nursing talent, where the contradiction between supply and demand is more pronounced (Nes et al., 2023; Vanckaviciene et al., 2024). Relevant studies show that (Romem and Rozani, 2024) some nursing master’s degree graduates tend to leave the clinical frontline or turn to non-nursing fields in their career planning, and their risk to the industry is much higher than that of undergraduate-level talent. If this trend continues, it will impede the sustainable development of nursing and threaten public health and social well-being. This situation underscores the urgent need for effective strategies to develop and retain a high-level nursing workforce, particularly among MNS students.

The concept of Future Self-Continuity (FSC) was first proposed by Ersner-Hershfield et al. (2009) and refers to an individual’s perception of the consistency and continuity between the present and future selves over time (Wang and Chen, 2020). In recent years, research on FSC has increased. Relevant studies have shown that (Chen, 2021; Fennell et al., 2019; Li, 2019; Yang and Zeng, 2022) individuals with high levels of FSC exhibit lower academic procrastination, higher learning engagement, more outstanding academic performance, a stronger sense of social responsibility, and a more positive attitude toward life. The theory of temporal self-evaluation posits that individuals continuously assess the connection and divergence between their present and future selves (Sedikides et al., 2023). This temporal self-evaluation directly influences motivation, goal setting, and behavioral choices. Self-concept theory further indicates that FSC is a crucial dimension in the development of professional self-concept (Bi et al., 2023). By integrating present experiences with future visions, individuals form a coherent professional identity, thereby guiding career persistence and commitment. For Master of Nursing Specialists (MNS), the level of FSC is directly related to their future professional identity and long-term adherence to the nursing industry. Relevant studies (Afshar et al., 2021) have confirmed that an individual’s clear understanding of their future self and a positive association with it can significantly enhance their professional commitment and engagement. This kind of professional commitment and investment is the core driving force for developing high-level nursing talent and is also key to alleviating the current shortage of high-level talent in the nursing industry and promoting the industry’s professional upgrading. Improving the FSC of the MNS community has a significant positive impact on the high-quality, sustainable development of the nursing industry.

Social support refers to the various material and spiritual support and help that individuals receive from social networks, which come from a wide range of sources, including emotional comfort provided by family, academic guidance, and resource guarantee provided by school, and experience sharing and encouragement among peers (Zell and Stockus, 2025). Previous studies have demonstrated that a significant positive correlation exists between social support and FSC, and that higher levels of social support strengthen FSC (Xue et al., 2023). From a theoretical perspective, social capital theory views social support as a crucial form of capital embedded within social networks, providing key pathways for individuals to access resources, information, and opportunities essential for career development (Lv et al., 2023). For Master of Nursing Science (MNS) students transitioning from academic learning to clinical practice, such support networks play a central role in facilitating the acquisition of professional skills, socialization into careers, and career decision-making (McClunie-Trust et al., 2024). Furthermore, the stress-buffering hypothesis posits that social support can serve as a protective mechanism, mitigating the negative impact of stressors on mental health and career development (Acoba, 2024). Particularly during clinical placements, when MNS students face significant professional challenges and role transitions, robust social support can buffer the decline in career commitment and deterioration of future orientation caused by stress, thereby helping maintain the stability of their career trajectories (Gong et al., 2025). Throughout this process, social support serves as a vital bridge connecting individuals with their families, schools, and peers. By providing emotional care, informational resources, and practical assistance, it not only alleviates psychological stress but also enhances their understanding of the connection between current learning behaviors and future career goals. This, in turn, fosters the development of their future self-continuity (Ma et al., 2024).

Although existing research has demonstrated a correlation between social support and FSC (Xue et al., 2023), most studies rely primarily on cross-sectional surveys (Ma and Song, 2023; Xue et al., 2023), making it difficult to infer causal relationships and temporal effects. Furthermore, research has predominantly focused on the influence of early factors, such as individual characteristics and parental nurturing styles, on FSC (Shen et al., 2022; Shen et al., 2023), yet has failed to fully elucidate the mechanisms underlying its dynamic changes during critical career development periods. This is particularly true for MNS students, who occupy a unique transitional phase between academic study and clinical practice. Therefore, grounded in Schlossberg’s Transition Theory (Anderson et al., 2011), this study defines the MNS clinical practicum as a three-stage career transition trajectory: “Moving In-Moving Through-Moving Out”. Employing a longitudinal tracking design to systematically examine the developmental patterns of FSC among MNS. It also delves into the dynamic mechanisms of social support and FSC across different transition phases, aiming to provide empirical evidence to enhance professional identity and stability of retention among high-level nursing talent.

**Figure 1:**
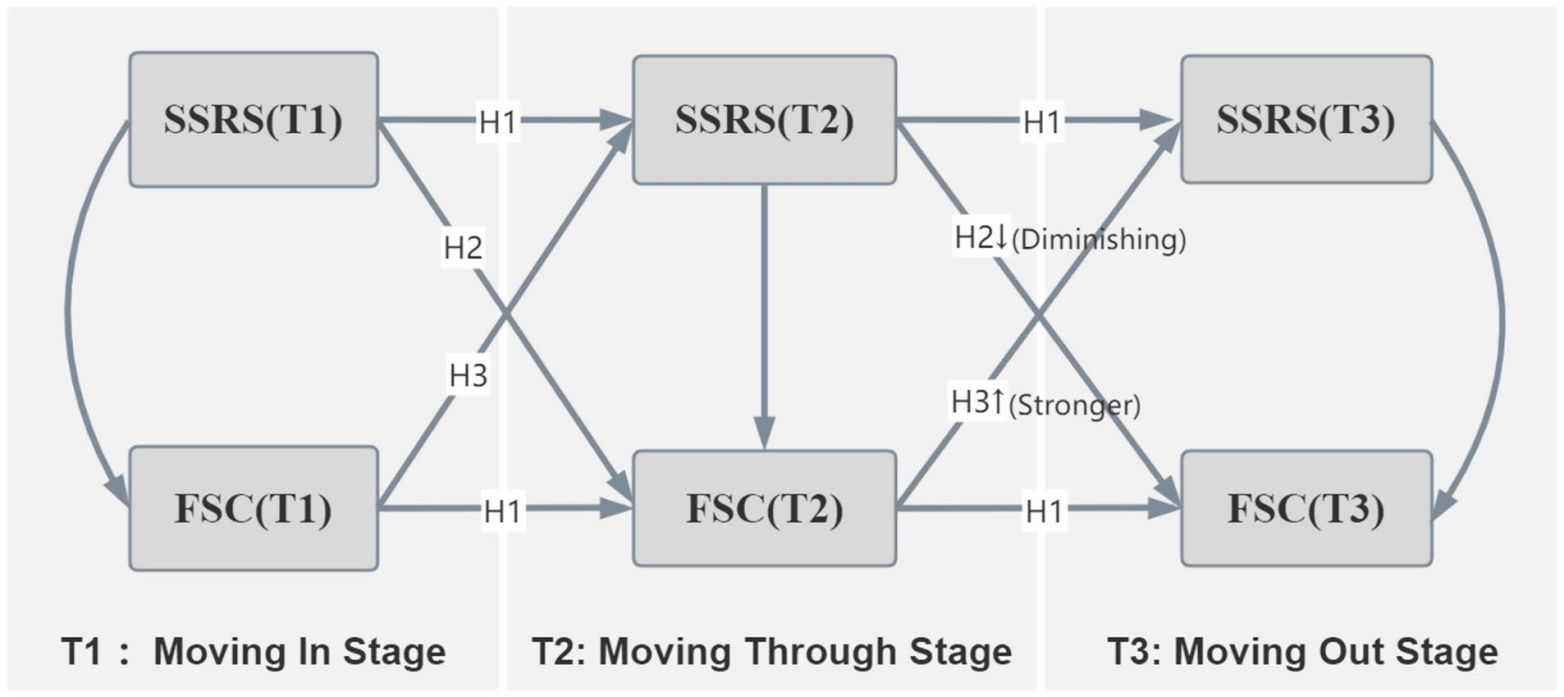
Conceptual model. SSRS, social support; FSC, future self-continuity.

Based on the aforementioned theoretical framework and practical context, this study proposes the following hypotheses (see Figure 1):

*H1*: During the MNS training period (T1-T3), both social support and FSC demonstrated significant temporal stability.

*H2*: Social support demonstrates a positive longitudinal association with FSC during both phases, but this effect gradually diminishes during the clinical internship phase (T2–T3).

*H3*: FSC demonstrated a positive longitudinal association with subsequent social support in both phases, with this effect being more pronounced during the clinical training phase.

## 2 Population and method

### 2.1 Research population

This study employs a longitudinal design and recruits participants through multi-center convenience sampling. Between September 2023 and December 2025, the research team established connections with multiple medical schools offering MNS programs and their affiliated clinical teaching bases in Hangzhou, Ningbo, Wenzhou, and other cities within Zhejiang Province. Following formal approval from graduate administration offices and nursing departments at affiliated hospitals, researchers conducted unified recruitment sessions during new-student orientation periods. These sessions systematically outlined the study objectives, three-year follow-up design, voluntary participation principle, data confidentiality measures, and participants’ right to withdraw at any time without conditions. Interested students voluntarily signed written informed consent forms after thoroughly reviewing the study materials, asking questions, and receiving answers. They then completed baseline questionnaires. All participants were explicitly informed that their decision to participate or withdraw would have no bearing on their academic performance or internship evaluations.

Inclusion Criteria: (1) Full-time MNS graduate students with formal enrollment status; (2) Voluntary participation in this study and signing of informed consent. Exclusion Criteria: (1) History of mental or psychological disorders (confirmed by hospital diagnosis); (2) Being on leave of absence, withdrawn, or in other abnormal enrollment status during the study period; (3) Individuals requesting withdrawal during the study. Referencing previous research (Lennon et al., 2018), to ensure the accuracy of structural equation modeling identification, the sample size must be ≥200 cases. To address the complexity of CLPM and LGCM, an *a priori* power analysis was also conducted. Based on prior research indicating moderate correlations between social support and FSC (*r* ≈ 0.30 ~ 0.45) (Xue et al., 2023), anticipated cross-lagged effects of *β* ≈ 0.15 ~ 0.20 were estimated. Monte Carlo simulation-based power analysis for longitudinal SEM (Muthén and Muthén, 2002) indicated that a sample size of 200 ~ 250 achieves 80% power at *α* = 0.05 for effects of this magnitude. With 5% attrition, 270 participants were recruited, yielding a final analytic sample of 263 that meets both requirements.

### 2.2 Data collection and quality control

This study employs a longitudinal tracking design, guided by Schlossberg’s Transition Theory framework. Through expert consultation and research group discussions, three theoretically significant time points were identified for data collection. The first survey (T1) was completed within the first month of enrollment (September 2023), corresponding to the Moving In stage. At this point, students had completed initial adaptation and reached a stable baseline, facilitating accurate measurement of baseline FSC and social support levels while avoiding interference from initial emotional fluctuations upon enrollment. The second survey (T2) was conducted 1 month after the commencement of the clinical practicum (June 2024), corresponding to the Moving Through stage. By this point, students had preliminarily entered the clinical domain, confronting challenges and pressures from role transitions. This served as a critical window for observing the buffering effects of social support and fluctuations in FSC. The third survey (T3) is conducted at the conclusion of clinical internships (December 2025), corresponding to the Moving Out stage. By this point, students have completed 18 months of clinical practice and developed a relatively stable professional self-concept, enabling effective assessment of adjustments and reconstructions in professional self-concept following a full internship cycle. The intervals between the three surveys are 9 months and 18 months, respectively, covering the critical professional transition phases for MNS graduate students from enrollment to the end of their internships.

### 2.3 Survey tools

#### 2.3.1 General information questionnaire

The general information questionnaire was designed by the research team, including gender, age, ethnicity, marriage, first education, whether they are fresh graduates, length of service, place of origin, family income, whether they have siblings, student cadres, parents’ work, parents’ education, study and life pressure, etc.

#### 2.3.2 Future self-continuity questionnaire, FSCQ

The FSCQ was compiled by Sokol and Serper (2020) based on the theoretical structure of future self-continuity and was Sinicized and applied by Shen et al. (2022) in 2020 to measure individuals’ level of future self-continuity. Using the Likert 6-level scoring method, 1 ~ 6 points are assigned from “completely inconsistent with oneself” to “completely in line with oneself”, with a total score of 10 ~ 60 points; the higher the score, the greater the individual’s future self-continuity. The questionnaire has good reliability and validity, with Cronbach’s *α* coefficients ranging from 0.75 to 0.88 for each item (Zhang et al., 2022).

#### 2.3.3 Social support rate scale, SSRS

The SSRS was compiled by Xiao (1994) in 1986 to evaluate the level of social support among individuals; it comprises 10 items across 3 dimensions: subjective support, objective support, and support use. The scale ranges from 12 to 66 points, with higher scores indicating greater social support for the individual. A score of 22 points or less is considered low, 23 to 44 points is medium, and 45 to 66 points is high. The scale shows good reliability, with Cronbach’s α coefficients ranging from 0.825 to 0.896 for each item and the total score (Liu et al., 2008).

### 2.4 Statistical analysis

SPSS 27.0 and Mplus 8.11 software were used for data analysis, and the number of cases and percentages were used, and the mean *±* standard deviation was used for the mean data. Pearson correlation was used to analyze the correlation between variables. Intergroup comparisons use Harman’s one-factor test to assess common-method bias. If the variance explained by the first factor is less than 40%, no significant common method bias is considered present. Confirmatory factor analysis (CFA) was conducted at each wave to evaluate the construct validity of the SSRS and FSCQ, with model fit assessed using the same indices. Missing data were handled using full-information maximum likelihood (FIML) estimation under the missing-at-random (MAR) assumption. Before longitudinal analyses, longitudinal measurement invariance of the SSRS and FSCQ across the three measurement waves was examined sequentially using *MLR* estimation, by testing configural, metric (equal factor loadings), scalar (equal loadings and intercepts), and strict (equal loadings, intercepts, and residual variances) invariance models; to examine longitudinal changes in latent means, the latent means of both SSRS and FSCQ were fixed to 0 at T1 as the reference, while the latent means at T2 and T3 were freely estimated; between-wave residual covariances were specified for each item to account for item-specific method effects (Little, 2024). Measurement invariance was considered supported when Δ*CFI* ≤ 0.010 and Δ*RMSEA* ≤ 0.015 (Chen, 2007). Latent Growth Curve Modeling (LGCM) is used to determine the linear developmental trajectories of variables. First, a univariate LGCM was constructed for social support and future self-continuity. Then, a parallel development of the LGCM for social support and future self-continuity was constructed, and a cross-lagged panel model (CLPM) was used to analyze the temporal relationship between social support and future self-continuity. To control for potential confounding effects, demographic and psychosocial variables that demonstrated significant differences in the baseline univariate analyses were included as time-invariant covariates in both the LGCM and CLPM analyses. The model-fitting indices (Gao et al., 2023) are: *CFI* > 0.9, *TLI* > 0.9, *SRMR* < 0.08, and *RMSEA* < 0.08. The statistical significance level was set at *α* = 0.05 for all analyses.

## 3 Results

### 3.1 Common method bias test

All scales were completed via MNS self-report, with common method variance examined using Harman’s one-factor test. Results revealed nine factors with eigenvalues exceeding 1. The first factor explained 26.07% of variance, falling below the 40% critical threshold, indicating no significant common method variance in this study.

### 3.2 General characteristics of the study population

A total of 270 MNS graduate students were included in this study in T1, and 263 were included in T2, resulting in a total loss-to-follow-up rate of 2.59%. General information about the survey subjects is shown in Table 1. The means and standard deviations of SSRS and FSC across the three measurement waves are presented in Table 2. SSRS scores remained stable across the three time points, with no statistically significant difference. In contrast, FSC scores declined significantly over time, with post-hoc comparisons indicating that all three time points differed significantly from one another.

**Table 1:** General characteristics of the study population (*N* = 263).

Variable		*N*(%)	FSCQ (*±s*)	*t/F*	*P*
Gender	Male	33(12.5)	34.85 ± 13.51	0.137^a^	0.892
	Female	230(87.5)	34.52 ± 5.80		
Age	20–25	178(67.7)	34.49 ± 7.47	3.130^b^	0.03
	26–30	55(20.9)	35.15 ± 6.37		
	31–35	22(8.4)	36.18 ± 4.42		
	≥36	8(3.0)	27.63 ± 8.48		
Grade	First year of graduate school	101(38.4)	37.48 ± 7.93	10.501^b^	<0.001
	Second year of graduate school	80(30.4)	33.57 ± 5.17		
	Third year of graduate school	82(31.2)	32.55 ± 5.92		
Marriage	Unmarried without lovers	120(45.6)	34.08 ± 6.39	2.184^b^	0.09
	Unmarried lovers	104(39.5)	35.61 ± 8.16		
	Married	36(13.7)	33.78 ± 6.56		
	Other	3(1.1)	27.00 ± 5.75		
First degree	College degree	48(18.3)	34.65 ± 5.34	0.008^b^	0.93
	Undergraduate	215(81.7)	34.54 ± 7.56		
Are you a fresh graduate or a past graduate	Fresh graduates	125(47.5)	33.59 ± 6.45	-2.097^a^	0.037
	Past graduates	138(52.5)	35.44 ± 7.73		
Birthplace	Rural	142(54.0)	33.89 ± 6.23	1.732^b^	0.179
	Town	67(25.5)	35.87 ± 7.03		
	City	54(20.5)	34.70 ± 9.36		
Whether you are an only child	Yes	102(38.8)	34.60 ± 9.52	0.056^a^	0.955
	No	161(61.2)	34.54 ± 5.24		
Whether you are a student cadre	Yes	186(70.7)	34.64 ± 6.98	0.270^a^	0.788
	No	77(29.3)	34.38 ± 7.72		
Learning pressure	No stress	11(4.2)	40.55 ± 17.17	5.270^b^	0.002
	Less stress	41(15.6)	36.12 ± 7.57		
	Moderate stress	103(39.2)	34.96 ± 5.52		
	Stressful	108(41.1)	32.98 ± 6.43		
Life stress	No stress	34(12.9)	38.09 ± 10.83	4.095^b^	0.007
	Less stress	105(39.9)	34.85 ± 5.61		
	Moderate stress	58(22.1)	33.48 ± 6.91		
	Stressful	66(25.1)	33.24 ± 6.89		

^a^Independent samples *t*-test; ^b^One-way ANOVA.

**Table 2:** Descriptive statistics and longitudinal comparison of SSRS and FSC across three measurement waves (*N* = 263).

Variables	T1	T2	T3	*F*	*P*	*η^2^ₚ*
SSRS	37.01 ± 4.82^a^	37.15 ± 4.95^a^	37.10 ± 4.78^a^	0.85	0.428	
FSC	39.05 ± 7.15^a^	37.42 ± 7.30^b^	34.28 ± 7.65^c^	45.62	<0.001	0.148

SSRS, social support; FSC, future self-continuity; Same letters indicate non-significant differences between time points; letters are arranged in descending order according to mean values.

### 3.3 Pearson correlation analysis

**Figure 2:**
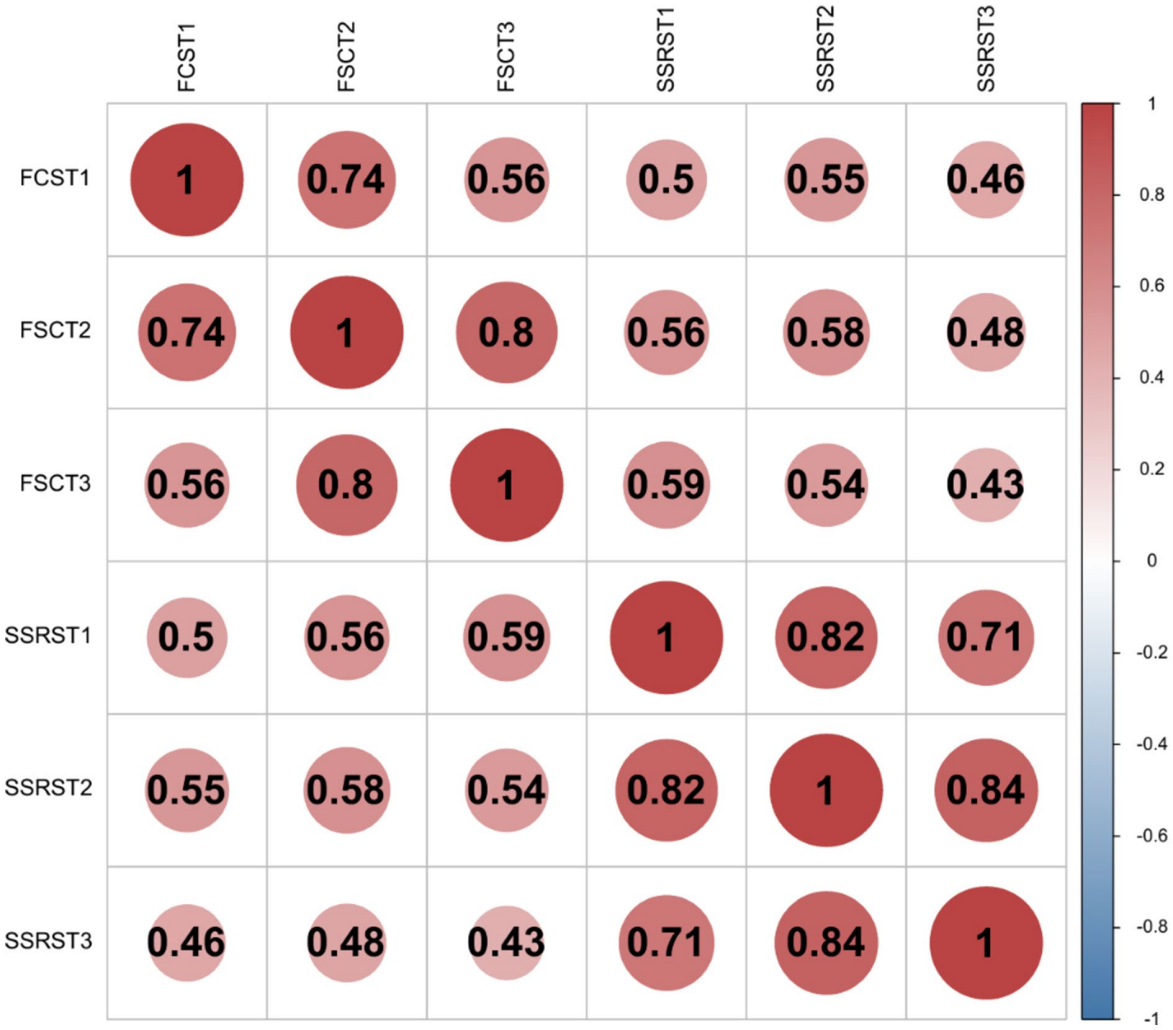
Correlation visualization heatmap. SSRS, social support; FSC, future self-continuity.

Correlation analysis revealed a significant positive correlation between social support and FSC of MNS graduate students at each time point (T1, T2, and T3). The correlation coefficients between each variable were statistically significant (*p <* 0.05) (see Figure 2 for details).

### 3.4 Reliability and validity analysis

Confirmatory factor analysis (CFA) and internal consistency reliability analysis were conducted for both the SSRS and FSCQ at each of the three measurement waves (T1–T3), with results presented in Table 3. Both scales demonstrated good model fit across all time points, with *χ^2^/df* < 2, *CFI* and *TLI* both exceeding 0.95, *RMSEA* < 0.08, and *SRMR* < 0.08, all of which satisfied the recommended criteria (Hu and Bentler, 1999). Standardized factor loadings for all items ranged from 0.72 to 0.91 across scales and waves, all exceeding the 0.60 threshold for acceptable indicator reliability. No modification indices exceeded 10, and no residual covariances were freed in any of the six models. Convergent validity was supported by average factor loadings above 0.80 and AVE exceeding the 0.50 threshold for both scales across all waves (Fornell and Larcker, 1981). Internal consistency was satisfactory, with *Cronbach’s α* ranging from 0.885 to 0.891 for the SSRS and from 0.893 to 0.902 for the FSCQ, all exceeding 0.80 (Nunnally, 1978). These CFA results served solely to establish psychometric validity prior to computing scale total scores; the LGCM and CLPM analyses were subsequently conducted using observed total scores as manifest variables, consistent with the original scale scoring conventions.

**Table 3:** Internal consistency and confirmatory factor analysis of the SSRS and FSCQ at each measurement wave.

Time point	Scale	*χ*^2^/df	CFI	TLI	RMSEA(95%CI)	SRMR	Average factor loading	AVE	Cronbach’s *α*
T1	SSRS	1.843	0.971	0.962	0.057(0.031 ~ 0.082)	0.048	0.807	0.652	0.887
	FSCQ	1.682	0.978	0.970	0.051(0.024 ~ 0.077)	0.043	0.823	0.677	0.896
T2	SSRS	1.927	0.968	0.958	0.060(0.035 ~ 0.085)	0.051	0.814	0.663	0.891
	FSCQ	1.754	0.975	0.967	0.054(0.028 ~ 0.079)	0.046	0.831	0.690	0.902
T3	SSRS	1.779	0.974	0.965	0.055(0.028 ~ 0.079)	0.046	0.804	0.647	0.885
	FSCQ	1.631	0.980	0.973	0.049(0.021 ~ 0.074)	0.041	0.818	0.669	0.893

SSRS, social support; FSC, future self-continuity.

### 3.5 Longitudinal measurement invariance

To ensure meaningful longitudinal comparisons, measurement invariance of the SSRS and FSCQ across T1, T2, and T3 was examined sequentially using MLR estimation (see Table 4). For both scales, the configural model demonstrated excellent fit, and imposing successive equality constraints on factor loadings (metric), item intercepts (scalar), and residual variances (strict) produced only negligible changes in model fit, with all Δ*CFI* and Δ*RMSEA* values well below the recommended thresholds (Chen, 2007). These results support strict longitudinal measurement invariance for both scales, indicating that the factor structure, factor loadings, item intercepts, and residual variances were equivalent across the three measurement occasions, justifying subsequent longitudinal comparisons in the LGCM and CLPM analyses.

**Table 4:** Longitudinal measurement invariance results for the FSCQ and SSRS across three measurement waves.

Model		*χ^2^*	*df*	*P*	CFI	TLI	RMSEA(90%CI)	SRMR	ΔCFI	ΔRMSEA
FSCQ	Configural	399.74	372	0.155	0.992	0.990	0.017(0.000,0.029)	0.047	–	–
	Metric	417.16	390	0.165	0.992	0.991	0.016(0.000,0.028)	0.049	−0.0001	−0.001
	Scalar	438.12	410	0.163	0.992	0.991	0.016(0.000,0.028)	0.051	0.0001	0.0001
	Strict	467.52	430	0.103	0.989	0.989	0.018(0.000,0.029)	0.052	−0.003	0.002
SSRS	Configural	405.85	372	0.109	0.994	0.993	0.019(0.000,0.030)	0.034	–	–
	Metric	418.07	390	0.157	0.995	0.994	0.017(0.000,0.028)	0.035	0.001	−0.002
	Scalar	437.50	410	0.168	0.995	0.995	0.016(0.000,0.028)	0.037	0.0001	−0.001
	Strict	446.95	430	0.277	0.997	0.997	0.012(0.000,0.025)	0.037	0.002	−0.004

ΔCFI and ΔRMSEA represent the changes in these measures for each model compared to the configural model.

### 3.6 Analysis of the development trajectory of SSRS and FSC

Construct a univariate LGCM for MNS graduate SSRS and FSC. On this basis, construct an LGCM for a parallel development model. Given that the time intervals between the three measurements were 9 months and 18 months, constituting a non-equidistant longitudinal design, this study followed recommendations from prior research (Ram and Grimm, 2007). When constructing the latent variable growth model, the slope factor loadings for the three measurements were set to 0, 1, and 3, respectively, to accurately reflect the impact of actual time intervals on developmental trajectories. The model intercept represents the initial levels of MNS graduate SSRS and FSC, and the slope represents the developmental rate for each period.

#### 3.6.1 The trajectory of social support

**Figure 3:**
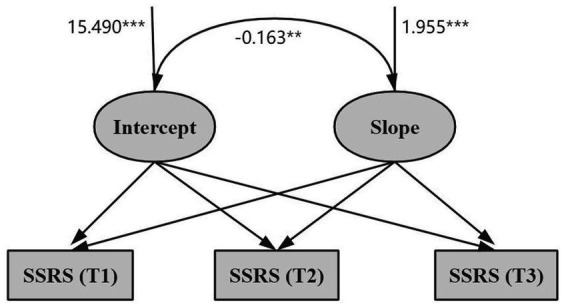
Latent variable growth model of SSRS. ***p < 0.001, **p < 0.05.

Construct an unconditional linear latent variable growth model for social support of MNS graduate students, with model fit indices results of *χ^2^* = 1.531, *CFI* = 0.967, *TLI* = 0.978, *RMSEA* = 0.045, *SRMR* = 0.012, indicating excellent fit. The model intercept, representing the initial level of social support, was 36.990 (*p* < 0.001), and remained stable over the 3 measurement occasions (*s* = 0.03, *p* = 0.752). Both the intercept (*σ^2^* = 15.490, *p* < 0.001) and slope (*σ^2^* = 1.955, *p* < 0.001) showed significant differences in variability. This suggests significant inter-individual differences in the initial level and rate of SSRS development among MNS graduate students. The intercept and slope were significantly negatively correlated (*r* = −0.163, *p* = 0.027), indicating a significant negative relationship between the initial level of SSRS and the rate of decline in social support among MNS graduate students; the higher the initial level of SSRS, the slower the rate of decline. Figure 3 shows the model results.

#### 3.6.2 The trajectory of FSC

**Figure 4:**
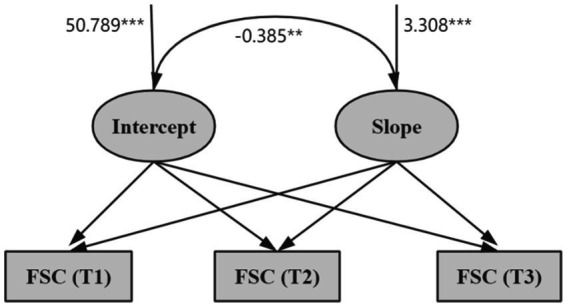
Latent variable growth model of FSC. ***p < 0.001, **p < 0.05.

Construct an unconditional latent variable linear growth model for MNS graduate FSC, with model fit indices results of *χ^2^* = 1.822, *CFI* = 0.979, *TLI* = 0.966, *RMSEA* = 0.056, *SRMR* = 0.026, indicating excellent fit. The intercept, representing the initial level of FSC, was 39.026 (*p* < 0.001), indicating a declining trend across the three measurements (*s* = −1.576, *p* < 0.001). Both the intercept (*σ^2^* = 50.789, *p* < 0.001) and slope (*σ^2^* = 3.308, *p* < 0.001) exhibited significant variability, suggesting significant inter-individual differences in the initial level and rate of change of MNS graduate FSC. The intercept and slope were significantly negatively correlated (*r* = −0.385, *p* < 0.001), indicating a negative relationship between the initial state and the rate of change in future self-continuity among MNS graduates. Higher initial FSC levels among MNS graduates are associated with slower decline rates, as shown in Figure 4.

#### 3.6.3 Parallel latent variable growth model of social support and FSC

**Figure 5:**
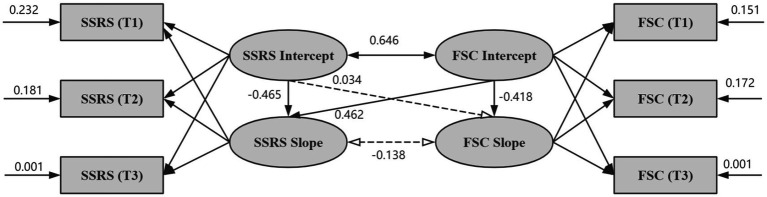
MNS graduate social support and FSC parallel latent variable growth model.

The LGCM of the parallel development model of social support and FSC for MNS graduate students was constructed to investigate the dynamic relationship between the two. During the initial unconstrained estimation of this model, minor negative residual variances (Heywood cases) (Kolenikov and Bollen, 2012) were observed for SSRS and FSC at T3. As these estimates were non-significant, we followed standard structural equation modeling practices and constrained these residual variances to a near-zero positive value (0.001) to ensure parameter admissibility (Dillon et al., 1987; Kolenikov and Bollen, 2012). This necessary constraint yielded a fully admissible solution without substantially altering the overall model fit or structural path estimates. After adjusting for the aforementioned covariates (age, grade, fresh/past graduate status, learning pressure, and life stress), the model fit was excellent: *χ*^2^ = 10.714, *CFI* = 0.967, *TLI* = 0.973, *RMSEA* = 0.045, and *SRMR* = 0.030. The initial level of social support was longitudinally associated with its rate of change (*β* = −0.465, *p* < 0.001), and the initial level of FSC was also longitudinally associated with its rate of change (*β* = −0.418, *p* < 0.001). Secondly, there was a significant positive correlation between the initial levels of social support and FSC (*β* = 0.646, *p* < 0.001), whereas the correlation between changes was not statistically significant (*β* = −0.138, *p* = 0.321). The initial level of social support was not longitudinally associated with the rate of change of FSC (*β* = 0.034, *p* = 0.726), while the initial level of FSC was longitudinally associated with the rate of change of social support (*β* = 0.462, *p* < 0.001). The parallel latent variable growth model is shown in Figure 5.

### 3.7 Cross-lagged model analysis

**Figure 6:**
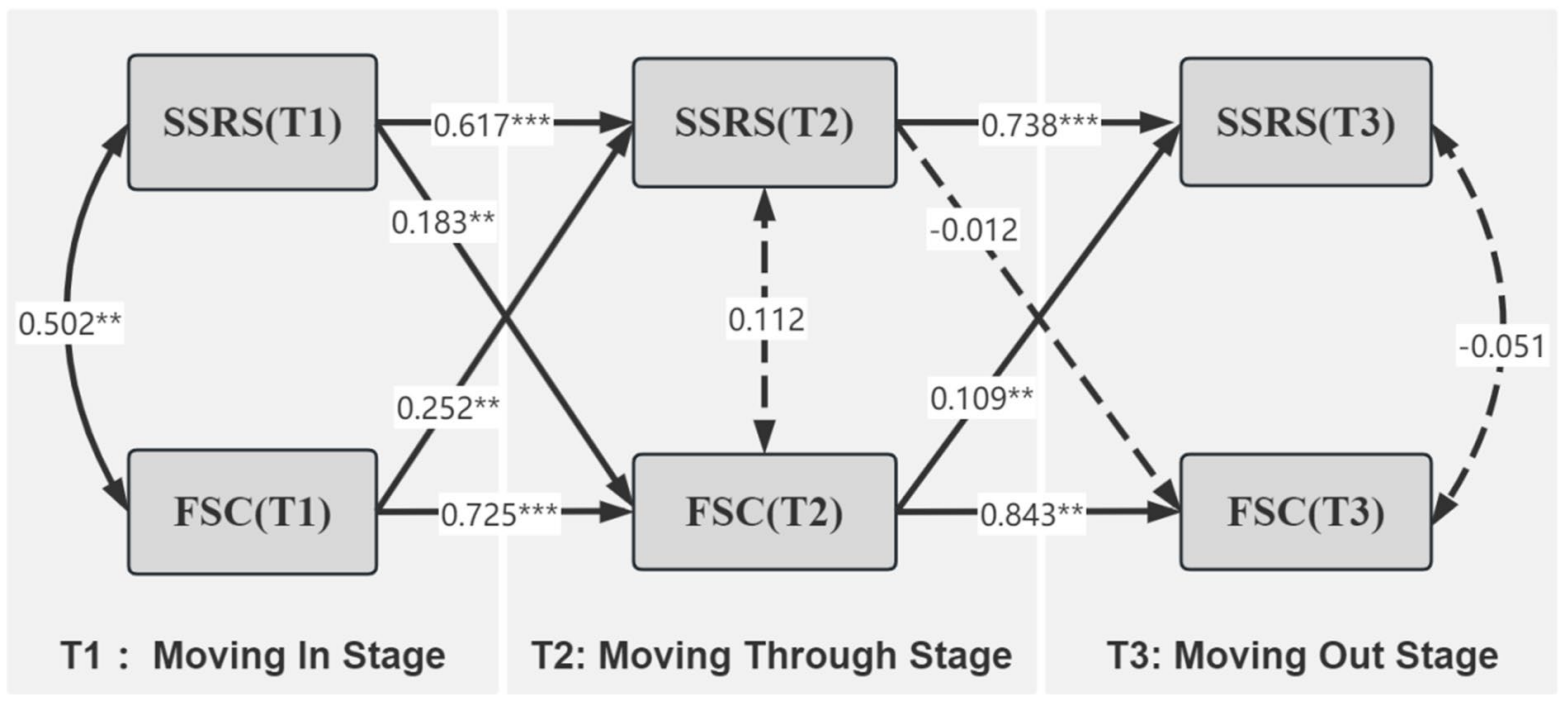
Cross-lag model of social support and FSC for MNS graduate students. Double arrows represent correlations, solid lines represent significant paths, and dashed lines represent non-significant paths. All path coefficients are standardized. ***p < 0.001, **p < 0.05.

To further investigate the asymmetric longitudinal relationship between social support and FSC and to strengthen the argument for temporal sequencing, a cross-lagged structural equation model was constructed. After controlling for significant demographic and stress-related covariates, the model fit results were *χ*^2^ = 6.029, *CFI* = 0.965, *TLI* = 0.947, *RMSEA* = 0.044, and *SRMR* = 0.020, indicating a better fit. There were statistically significant path coefficients from FSC to social support in T1–T2 and T2–T3 (T1–T2: *β* = 0.252, *p* = 0.008; T2–T3: *β* = 0.109, *p* = 0.034). The above results suggest that FSC demonstrated temporal precedence over social support levels in the subsequent stage. The path coefficient from social support to FSC was statistically significant in the T1–T2 period (*β* = 0.183, *p* = 0.015), and there was no statistically significant difference (*β* = −0.012, *p* = 0.842) in the T2–T3 period. The above results suggest that social support has a temporal precedence over FSC, but the longitudinal association effect on FSC disappears as the practice progresses. The model accounted for substantial variance in all endogenous variables: *R*^2^ = 0.584 for SSRS at T2, *R*^2^ = 0.685 for FSC at T2, *R*^2^ = 0.572 for SSRS at T3, and *R*^2^ = 0.713 for FSC at T3 (all *p* < 0.001), indicating that the model possessed good explanatory power across both transition phases. The longitudinal pathways of social support and FSC in MNS graduate students at three time points are shown in Figure 6.

It is important to acknowledge a methodological consideration regarding the cross-lagged panel model employed above. The traditional CLPM estimates population-averaged associations between variables across time points but does not decompose between-person variance from within-person variance. As Hamaker et al. (2015) noted, this conflation can yield biased cross-lagged estimates when stable individual differences are present. In the current study, however, social support demonstrated near-zero growth across the three waves (*s* = 0.03, *p* = 0.752), indicating limited systematic within-person variation in this construct over the study period, which would constrain the additional explanatory yield of a random-intercept decomposition. Nonetheless, future studies incorporating more measurement waves and more heterogeneous samples are encouraged to apply the RI-CLPM framework to further clarify the within-person temporal dynamics between social support and FSC.

## 4 Discussion

Guided by Schlossberg’s Transition Theory, this longitudinal study examined the developmental trajectories of social support and future self-continuity (FSC) among Master of Nursing Specialist (MNS) students across three critical transition phases: Moving In (T1, enrollment), Moving Through (T2, early internship), and Moving Out (T3, internship completion). Using LGCM and CLPM, we identified distinct developmental trajectories and phase-dependent, asymmetric temporal relationships between these two constructs. Although prior longitudinal studies using CLPM have examined the association between social support and FSC among general university student populations, the phase-specific dynamics of this relationship across the structured clinical transition stages of MNS training had not been systematically characterized. The present findings extend the existing evidence base by revealing how the directionality of the social support – FSC relationship shifts meaningfully across transition phases, offering context-specific insights into professional identity formation and talent retention in graduate nursing education.

### 4.1 Stability of social support across the transition trajectory

Consistent with Hypothesis 1, MNS students maintained a moderately stable level of social support throughout their three-year training period. This stability may be attributed to macro-level policy support for graduate nursing education and the sustained availability of institutional resources (McKenna et al., 2023). At the micro-level, while mentorship and teamwork are core facilitators of social support (Stockard et al., 2021), clinical faculty face multi-tasking pressures that limit their capacity to dedicate sufficient time to providing personalized guidance to MNS students (Leep Hunderfund et al., 2022). Furthermore, competitive dynamics within research teams may constrain emotional support and resource sharing (Wiesenthal et al., 2023). Concurrently, the school’s career guidance lacks in-depth support for clinical research positions and career planning tailored to the professional characteristics of MNS graduate students, resulting in insufficient systematic support for their career development (Traynor et al., 2025). In summary, micro-level social support remains relatively stable in terms of basic security but falls short in meeting personalized and in-depth needs. This imbalance contributes to the overall perception of moderate and relatively stable social support among MNS graduate students.

Notably, the negative correlation between initial social support and its rate of change (*r* = −0.163, *p* = 0.027) indicates that students with higher baseline support levels experience slower declines in support over time. From a positive psychology perspective, early perceived support fosters resilient belief systems (Zell and Stockus, 2025), enabling students to navigate challenges more effectively and proactively build social resources, such as professional networks and clinical mentorship, thereby buffering against later support decline (Wu et al., 2024). This aligns with findings from McClunie-Trust et al. (2024), who found that nursing students who established industry connections during clinical placements strengthened their professional identity. These findings underscore the critical importance of establishing robust support systems early in training, particularly during the “adaptation phase” when students are most receptive to external resources.

### 4.2 Declining FSC and the role of initial levels

In contrast to the stability of social support, FSC exhibited a significant and sustained decline across the three measurement rounds (*s* = −1.576, *p* < 0.001), thus only partially supporting Hypothesis 1. While the stability of social support aligned with our prediction, the declining trajectory of FSC was not anticipated, suggesting that the psychological demands of clinical transition exert a more erosive effect on future-oriented self-perception than on perceived external support (Tang et al., 2025). This asymmetry between the trajectories of social support and FSC represents a theoretically meaningful finding: external support systems may be maintained through institutional structures (Vabo et al., 2022), whereas FSC, being a more internally constructed psychological state, is more vulnerable to the identity disruptions characteristic of the Moving Through and Moving Out phases (Zhao et al., 2024). This decline theoretically corresponds to the challenges of the “transition phase,” where students must confront the realities of clinical practice, role ambiguity, and heightened performance pressure. During this phase, students may experience anxiety and self-doubt, undermining their ability to establish coherent connections between current efforts and future professional identity (Zhang et al., 2024). Simultaneously, as novices, they face scrutiny from patients and families, triggering fundamental questions about the value of their profession (Sessions and Pychlau, 2024), while exposure to adverse medical events further erodes their professional confidence (McCarrick et al., 2022). Furthermore, intense competition among highly educated nurses for desirable positions may exacerbate career uncertainty during the “breakthrough period,” thereby leading to a persistent decline in career security (Walker et al., 2019).

Importantly, the negative correlation between initial FSC and its rate of change (*r* = −0.385, *p* < 0.001) indicates that students with higher baseline FSC experienced a slower decline. This protective effect may stem from higher initial FSC levels in MNS, which often correlate with greater psychological resilience (Yi et al., 2024), enabling these individuals to sustain goal-directed behavior and adapt more effectively to clinical challenges. Students with a robust FSC are better able to recognize the relevance of current learning to future career goals, sustaining academic engagement and career commitment even under stress (Sedikides et al., 2023). These findings underscore the critical need for early interventions to strengthen FSC before students enter high-stress clinical settings.

### 4.3 Longitudinal relationships: FSC as a sustained antecedent of social support

By constructing a latent-variable growth model of social support combined with FSC, it is found that the initial level of FSC has a positive longitudinal association with the rate of change in social support, whereas the initial level of social support does not show a significant longitudinal association with the rate of change in FSC. One possible explanation is that MNS graduate students with higher initial FSC levels have a clearer career development picture and goal orientation, which prompts them to actively seek various social support resources, including actively contacting clinical tutors and participating in academic exchange activities, thus forming a positive accumulation of support resources (Chishima and Wilson, 2021). At the same time, research by Talluri et al. (2025) showed that MNS graduate students with high initial FSC levels showed stronger integration skills when faced with support resources. They can not only identify the advantages of different support subjects but also form support synergy through cross-group linkage, enabling scattered support resources to form a closed loop and further accelerating the expansion and deepening of the social support network. The initial level of social support is not longitudinally associated with the rate of change in FSC, and this may be because there is an essential difference between the “passive access” of social support and the “active development” of FSC. Even if individuals receive more social support in the initial stage, without clear goal orientation and intrinsic drive, this support may remain superficial, leaving these resources unable to be converted into motivation for FSC improvement (Zell and Stockus, 2025). More importantly, the improvement of FSC inherently depends on the individual’s self-worth, control over career path, and psychological resilience in coping with setbacks (Wang et al., 2025), and the formation of these intrinsic traits is more a result of self-exploration and goal practice than of the simple superposition of external support. Even if the initial social support is small, as long as the individual has a high FSC level, the lack of external support resources can be compensated through initiatives. On the other hand, if there is no internal goal drive, no amount of external support will stimulate individuals to continuously improve their FSC. This suggests that schools and hospitals need to foster “internal drive” and “external support” when training MNS graduate students, but the core remains cultivating students’ awareness of self-development.

### 4.4 Phase-specific effects of social support on FSC

The results of the cross-lag model show that FSC has a significant positive longitudinal association with social support from enrollment to the end of the internship. This finding is consistent with McClunie-Trust et al. (2024). This may be because in terms of behavioral decision-making, individuals with high FSC levels are more aware of the possible impact of their behavior on the future, so they are more inclined to adopt responsible behavior patterns in daily learning and social interactions, actively maintain good relationships with mentors and classmates, and pay attention to accumulating and maintaining long-term effective social support networks (Wan et al., 2023). At the same time, high FSC individuals are more inclined to consider long-term interests rather than short-term gains and losses in intertemporal decision-making (Xue et al., 2023), which helps them better plan their lives and futures, and actively seek social support that matches these plans, providing a strong guarantee for future development and success. In addition, from the perspective of social cognition theory, individuals with high FSC have a higher degree of recognition of their professional roles (Wu et al., 2024), which makes it easier to obtain recognition and support from mentors and peers, forming a two-way interactive support network, which also explains that FSC can continue to show temporal precedence over the improvement of social support levels in the process of vertical development.

Social support has a longitudinal association with FSC from the beginning of enrollment through the start of the internship, but this association disappears from the start to the end of the internship. This may be because MNS graduate students are in the theoretical learning stage from enrollment to internship, and the construction of FSC relies more on systematic knowledge input and external guidance from tutors and schools, which directly promotes the formation of FSC by enhancing the sense of belonging and professional commitment (Wu et al., 2024). Geary and Xu (2022) also highlighted the importance of external factors in helping individuals develop career motivation and identity and in promoting the construction of FSC. When graduate students enter the internship stage, the focus of learning shifts to practical application and experience accumulation, and the construction of FSC relies more on internal resources such as self-initiative and occupational adaptability or self-efficacy, and the direct influence of social support is weakened or disappears (Bodinof Jachowski et al., 2025), which is consistent with Lee et al.’s (2021) research results that social support is a moderating variable of occupational resilience and professional identity, rather than a direct impact on FSC. At the same time, MNS graduate students face a heavier time burden and greater work pressure during the internship, which may dilute the protective effect of external social support on FSC formation (Leep Hunderfund et al., 2022). Wang et al.’s (2024) research also suggests that social support influences behavior through self-efficacy, and that self-efficacy in internships may rely more on personal experience than on external support, thereby undermining the indirect pathways of social support. This reflects the evolutionary characteristics of FSC in career development, relying on external support in the formative stage and turning to internal dynamics in the practical period. This prompts us to build a phased support system of “institutional guidance – clinical autonomy” to achieve a smooth transition from exogenous shaping to endogenously driven development and to promote the inflow of MNS talents into the medical and health system.

### 4.5 Implications for nursing education and clinical training

The present findings carry several theoretically grounded implications for the design of educational and clinical training programs for MNS students. Specifically, the cross-lagged panel results revealed that FSC consistently demonstrated temporal precedence over subsequent social support across both transition phases, whereas social support’s longitudinal association with FSC was significant only during the pre-clinical period and dissipated as the internship progressed. This directional asymmetry, coupled with the significant declining trajectory of FSC observed across the three measurement waves (*s* = −1.576, *p* < 0.001), indicates that FSC is simultaneously the more vulnerable construct and the more consequential factor associated with professional development among MNS students. Institutional efforts should therefore position FSC cultivation as the primary target of intervention, rather than treating social support enhancement as an end in itself.

During the enrollment phase (Moving In), the most pressing priority is to establish a strong FSC foundation before students encounter the identity pressures of clinical practice. Our data indicate that a higher baseline FSC was associated with a slower rate of subsequent decline (*r* = −0.385, *p* < 0.001), underscoring the protective value of early investment. Institutions should implement structured career orientation programs that explicitly link academic learning to long-term professional trajectories. Crucially, mentorship relationships should be established during enrollment rather than upon clinical entry, as early mentoring that actively scaffolds professional identity formation rather than merely delivering informational support has been shown to exert the most durable influence on professional self-concept development (Krishna et al., 2025; Yao and Wu, 2025).

As students progress into the early internship phase (Moving Through), social support ceases to exert a significant direct effect on FSC, rendering resource-focused interventions insufficient. Attention should instead shift toward cultivating students’ internal psychological resources. Self-efficacy and occupational adaptability have been identified as core mediators of clinical competence development during internship (Bresolin et al., 2024), and clinical preceptors should be equipped to provide autonomy-supportive guidance that balances structured supervision with opportunities for self-directed problem-solving. Psychological resilience training integrated into clinical curricula rather than offered as ancillary support may be particularly effective for sustaining FSC when external support pathways are attenuated (Xiang et al., 2024).

During the internship completion phase (Moving Out), the sustained longitudinal association between FSC and social support suggests that students with maintained FSC continue to actively expand professional networks. Institutions can leverage this dynamic through alumni engagement and structured peer networking, while individualized career counseling grounded in each student’s career uncertainties can translate accumulated clinical experience into coherent professional goal structures (Talluri et al., 2025).

Taken together, these phase-specific findings call for a reconceptualization of MNS support systems: from a model that delivers support to students, toward one that cultivates students’ capacity to seek and sustain support through FSC development, a shift grounded in the empirical reality that the FSC and social support relationship is dynamically modulated across developmental contexts.

## 5 Limitations

Several limitations should be acknowledged. First, all participating institutions are located in Zhejiang Province, China. This single geographic sample may limit the generalizability of findings to master’s-level nursing cohorts trained within different healthcare systems or educational policy contexts. Future research should replicate this design across broader geographic regions and diverse institutional types. Second, although the cross-lagged panel model supports temporal precedence, causality cannot be fully established due to potential unmeasured confounders. Incorporating randomized components in future designs would strengthen causal inference. Third, the traditional CLPM conflates between-person stable trait variance with within-person temporal dynamics, potentially inflating cross-lagged estimates (Hamaker et al., 2015). Given the substantial intercept covariance between social support and FSC observed in this study, the pre-clinical longitudinal association of social support on FSC, the weaker and more phase-restricted effect, is most susceptible to attenuation and should be interpreted with caution, whereas the sustained temporal precedence of FSC over social support is more likely to remain directionally robust. Future studies are encouraged to apply the RI-CLPM to more rigorously isolate within-person dynamics. Finally, the sample consisted exclusively of MNS graduate students; whether these dynamics apply to undergraduate nursing students, newly licensed nurses, or other health professions trainees remains unclear. Larger, more heterogeneous samples would also enable subgroup analyses by gender, specialty, and institution.

## 6 Conclusion

This study examines the development trajectory and dynamic relationship between social support and FSC among MNS graduate students, utilizing the latent variable growth model and the cross-lag model. The results showed that the overall social support among MNS graduate students was moderate and relatively stable. The FSC showed a downward trend, and the initial level of FSC was longitudinally associated with the change rate of social support; the higher the initial level, the slower the change rate, whereas the initial level of social support was not associated with the change rate of FSC. Cross-lag analysis shows that FSC has a longitudinal association with social support, whereas social support has only temporal precedence over FSC in the early stage, and this association disappears as the internship progresses. It is suggested that the social support system be strengthened when MNS graduate students are enrolled, and that a multi-level, personalized support system be built, laying the foundation for improving the FSC level and promoting the sustainable, high-quality development of the nursing field. This study supports a temporal sequence, but the direction of causality requires further validation through experiments or RI-CLPM designs.

## Data Availability

The raw data supporting the conclusions of this article will be made available by the authors, without undue reservation.
